# Effects of Shot Peening and Electropolishing Treatment on the Properties of Additively and Conventionally Manufactured Ti6Al4V Alloy: A Review

**DOI:** 10.3390/ma17040934

**Published:** 2024-02-17

**Authors:** Wojciech Okuniewski, Mariusz Walczak, Mirosław Szala

**Affiliations:** Department of Materials Engineering, Faculty of Mechanical Engineering, Lublin University of Technology, Nadbystrzycka 36D, 20-618 Lublin, Poland; wojciech.okuniewski@pollub.edu.pl

**Keywords:** additive manufacturing, shot peening, electropolishing, peening, titanium alloy, Ti-6Al-4V, mechanical properties, microstructure, surface engineering

## Abstract

This literature review indicates that the basic microstructure of Ti6Al4V is bimodal, consisting of two phases, namely α + β, and it occurs after fabrication using conventional methods such as casting, plastic forming or machining processes. The fabrication of components via an additive manufacturing process significantly changes the microstructure and properties of Ti6Al4V. Due to the rapid heat exchange during heat treatment, the bimodal microstructure transforms into a lamellar microstructure, which consists of two phases: α′ + β. Despite the application of optimum printing parameters, 3D printed products exhibit typical surface defects and discontinuities, and in turn, surface finishing using shot peening is recommended. A literature review signalizes that shot peening and electropolishing processes positively impact the corrosion behavior, the mechanical properties and the condition of the surface layer of conventionally manufactured titanium alloy. On the other hand, there is a lack of studies combining shot peening and electropolishing in one hybrid process for additively manufactured titanium alloys, which could synthesize the benefits of both processes. Therefore, this review paper clarifies the effects of shot peening and electropolishing treatment on the properties of both additively and conventionally manufactured Ti6Al4V alloys and shows the effect process on the microstructure and properties of Ti6Al4V titanium alloy.

## 1. Introduction

**Figure 1:**
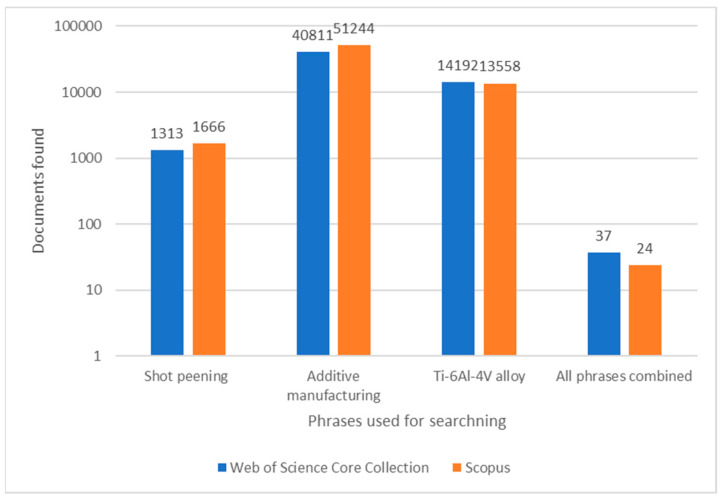
Search results for selected phrases related to this paper’s scope. The search was performed in the title, abstract and keywords of papers published in the years 2019–2023 and indexed in Web of Science Core Collection and Scopus.

Nowadays, the demand for improving materials from titanium alloys has increased tremendously. In comparison to structural steels or stainless steels, titanium alloys are characterized by a high strength-to-density ratio, corrosion resistance and sufficient operational performance with wide applications in industries such as aerospace, marine, automobile, biomedical, petrochemical, energy and many other industries [1,2,3]. As evidence, Figure 1 shows results from the research centers of Web of Science and Scopus and the number of articles on the subjects of shot penning, additive manufacturing and Ti-6Al-4V alloy. Combining phrases (as shown in Figure 1) significantly narrowed the field of the literature search. As can be read from Figure 1, additive manufacturing is a prominent subject of today’s studies. In addition, it is worth noting that these numbers only include the additive manufacturing phrase. There are definitely more articles that use specific AM terminology like SLM and DMLS or their full names without mentioning the phrase additive manufacturing or the AM abbreviation. Also noteworthy is the fact that there are numerous studies about shot peening treatment, which means that this type of surface treatment is still to be fully exploited. Moreover, it can be observed that even though recent reports suggest an increased interest in new β-phase titanium alloys, Ti-6Al-4V titanium alloy is continuously considered as a valuable subject of research.

Increased interest in titanium alloys is caused by many factors. Initially, Ti-6Al-4V alloy proved itself for the aerospace industry. Its previously mentioned advantages have turned out to be suitable for jet engines, gas turbines and many airframe components in aircraft applications [2,3,4]. However, its properties started to be utilized in other applications as well. For instance, the usage of Ti-6Al-4V alloy can be seen in medical applications, as world societies are focusing more on their health and starting to be more active. That results in much younger patients who do not want to change their lifestyle and place more physical strain on the implants. This means that implants have to serve longer with more efficiency [5]. The quality of implants is crucial because wear and corrosion processes contribute negatively to the properties of the implant, which may have a straight negative impact on the periprosthetic environment [6,7]. Table 1 shows a comparison of commonly applied materials in the biomedical industry in terms of their physicomechanical properties.

Titanium alloys are vulnerable to surface integrity, which is correlated to their performance. This generates the need for surface treatment of Ti-6Al-4V manufactured components as a way of improving surface integrity. The well-established post-producing surface treatment technologies for modifying the surface layer of Ti-6Al-4V are shot peening and electropolishing [12,13]. Numerous studies indicate that combining those technologies applied to steel is profitable [14,15]. Unfortunately, there is a lack of studies presenting the hybrid peening and electropolishing treatment applied for Ti-6Al-4V titanium alloy. Moreover, there is no proper review article about the impact of additively manufactured Ti-6Al-4V titanium alloy on shot peening and electropolishing. Therefore, this paper fills this gap in knowledge and compares the effect of surface treatments of conventionally manufactured titanium alloy with that of additively manufactured titanium alloy on its microstructure and properties. 

This review paper aims to clarify the effects of shot peening and electropolishing treatment on the properties of both additively and conventionally manufactured Ti6Al4V alloys. This paper synthesizes data about the influence of shot peening and electropolishing processes on the microstructure and properties of Ti6Al4V titanium alloy.

## 2. Manufacturing Methods for Ti6Al4V Alloys

The paper’s idea consists of Ti6Al4V alloy characterization, a presentation of conventional and additive manufacturing technologies, and consideration of the post-processing of the surface layer of Ti6Al4V components with special attention to shot peening and electropolishing. All issues are discussed to clarify the properties and microstructure development of the Ti6Al4V surface layer due to peening and electropolishing. The selection of literature was conducted on the basis of keywords such as Ti-6Al-4V, additive manufacturing, shot peening and electropolishing, as well as the work of our research team on this matter. The conducted review allows for identifying knowledge gaps and presenting potential future research trends. 

### 2.1. Material—Ti6Al4V Alloy Characterization

The subject of this review is Ti6Al4V alloy. The typical microstructure of this titanium alloy can be structurally categorized by the size and organization of dual α and β phases. Titanium alloys usually undergo thermomechanical treatments [16]. Ti–6Al–4V contains 6% aluminum element, which stabilizes the hexagonal close-packed α phase, and 4% vanadium element, which stabilizes the β phase. Both phases i.e., the α phase (hcp) and β phase (bcc), coexist at room temperature [17]. The nominal chemical composition of Ti6Al4V alloy is presented in Table 2.

**Figure 2:**
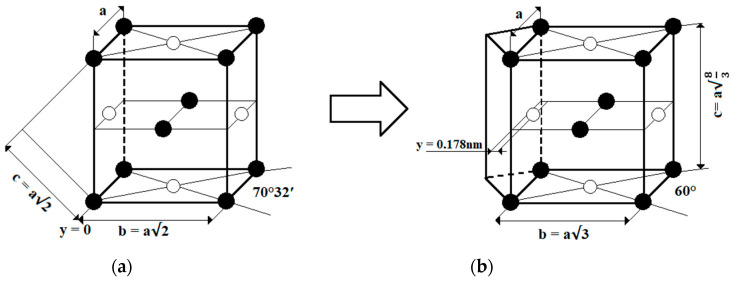
Martensitic transformation of unit cells in Ti6Al4V: β phase (**a**), α and α′ phases (**b**) based on [19,20].

The control and the optimization of the microstructure of Ti-6Al-4V alloy is one of the most important issues in achieving desired properties. During the heating process, the α phase transforms into the β phase approximately at a temperature of ~1000 °C, which is called the β transus temperature (*T_β_*). Heat treatment below β transus is referred to as sub-transus, whereas that above β transus is referred to as super-transus [18]. The cooling rate defines the transformation of β into → α (α′) (α′′). The transformation of unit cells is shown in Figure 2.

**Figure 3:**
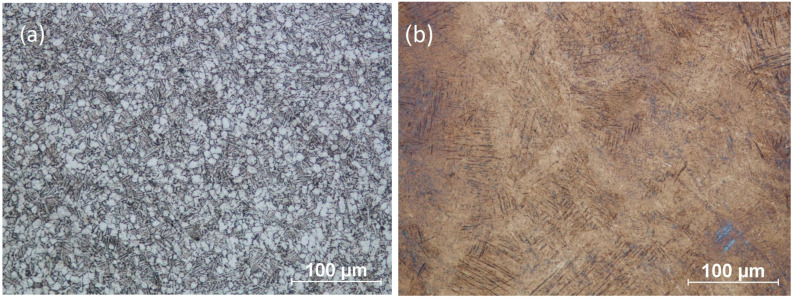
Micrographs of the microstructures of Ti-6Al-4V: (**a**) bimodal (α + β) microstructure of conventionally manufactured Ti-6Al-4V; (**b**) lamellar microstructure (martensitic microstructure) of Ti-6Al-4V additively manufactured via DMLS [21].

The basic microstructure of conventionally manufactured Ti6Al4V alloy is bimodal (α + β) with internally connected equiaxed primary α grains and lamellar transformed β grains. An example of this structure is shown in Figure 3a. On the other hand, a typical additively manufactured Ti6Al4V component shows a martensitic lamellar structure, which can be obtained as a Widmanstätten structure (Figure 3b) [21] or martensite plates.

**Figure 4:**
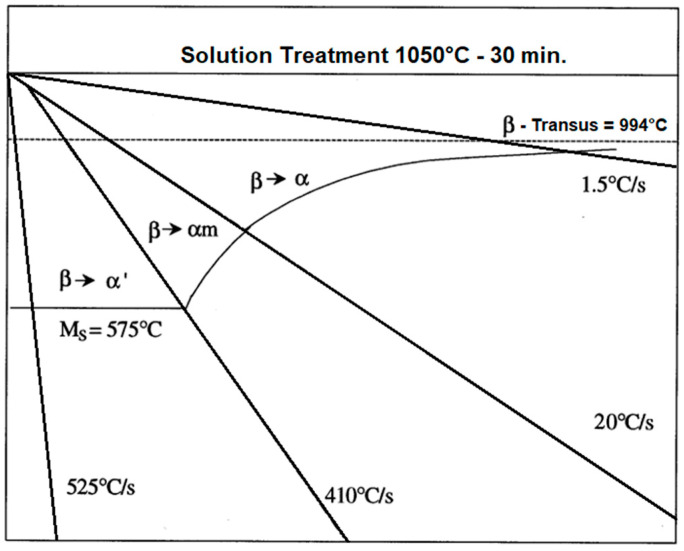
Schematic illustration of continuous cooling transformation for β-solution of Ti–6Al–4V titanium alloy treated at 1050 °C for 30 min [22].

The quenching temperature determines the hardened state of martensitic two-phase α + β alloys. The phase components might be in the form of martensitic precipitations of α′ and α_M_, along with grains of the metastable β_M_ phase [19]. The scheme of emerging phases depending on the cooling rate is shown in Figure 4.

The cooling medium also has an impact on the microstructure which then results in differences in Ti6Al4V properties. Table 3 describes the expected microstructure classified according to the cooling medium used and the phases in which the transformation occurs, irrespective of the material soaking time [23].

This literature review confirms that Ti-6Al-4V fabricated using conventional casting, plastic forming or machining methods has an α + β dual-phase microstructure. Additive manufacturing methods change the microstructure. Due to the rapid heat exchange during heat treatment, the bimodal microstructure transforms into a lamellar microstructure consisting of two phases: α′ + β (see Figure 3). 

### 2.2. Properties of Conventionally and Additively Manufactured Ti6Al4V Alloy

#### 2.2.1. Conventional Methods (CMs) for Ti6Al4V Alloy Fabrication

**Figure 5:**
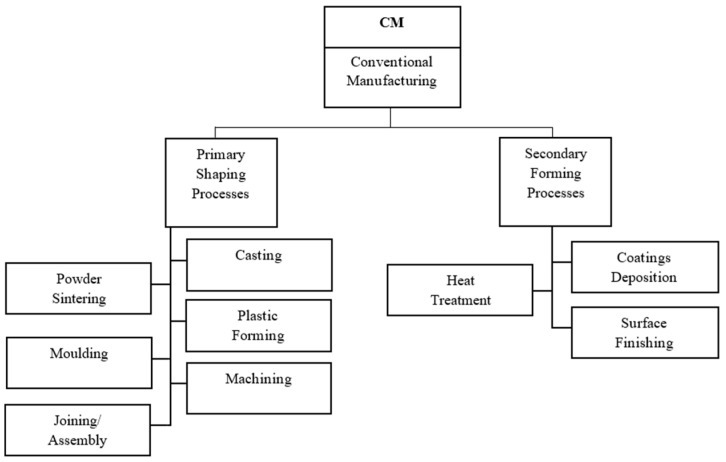
Conventional manufacturing methods based on [24,25].

The primary fabrication methods for titanium alloy components are casting technologies, plastic forming, joining and machining of as-received wrought bars, sheets, plates, etc. The basic classification of CMs is shown in Figure 5. These technological processes allow the specific design and properties of the component to be obtained, and these properties could be improved by heat treatment, surface engineering methods modification of surface layer properties or coating deposition. A comparison of the mechanical properties of conventionally processed Ti6Al4V alloy is shown in Table 4. 

Since obtaining a designed shape of detail is usually time-consuming when conventional manufacturing methods are used, the industry is systematically developing cost-effective manufacturing methods such as additive manufacturing. Components fabricated via conventional and additive manufacturing methods differ in microstructure, which implies the mechanical properties, operational performance and durability of Ti6Al4V structures. The main difference is that the microstructure of the Ti6Al4V consists of a titanium-based solid solution of α + β phases while additively manufactured components usually show a martensitic α′ + β microstructure; see Figure 3. In the case of traditionally fabricated Ti6Al4V components, the transformation of basic α + β into martensitic α′ + β can be achieved in conventional heat-treatment processes: quenching and annealing. In industry applications, the combination of solution heat treatment and aging operation is used in the aerospace and automobile industries [26]. The main reason is that the microstructure consists of a soft α phase and a β grain boundary [27]. 

**Table 4:** Mechanical properties of Ti6Al4V manufactured using conventional methods [28,29,30,31,32].

Processing of Ti6Al4V Alloy	Heat Treatment	Microstructure in Various Cooling Media				Ref.
		Microhardness	YS [MPa]	UTS [MPa]	A [%]	
Wrought (As-received)	Untreated	325 HV	880	960	14	[28]
	Untreated Ti6Al4V	190 HK	880	910	18	[29]
	water quenching + aging	320 HK	1110	1170	6.5	[29]
	Ti6Al4V air cooling + aging	210 HK	910	980	12.5	[29]
Forged	Mill annealed	-	1030	970	16	[30]
	Mill annealed	-	960	1006	18.4	[28]
Cast	-	330 HV	750	875	4.5	[31]
	-	-	865	980	13.5	[32]

However, the conventional methods (CMs) are systematically being replaced by additive manufacturing (AM) methods. The main reason for that is hard machining material which is caused by the low thermal conductivity. There are undeniable issues in refining, casting, forging or rolling leading to a notable amount of material waste, which in the long term leads to longer lead time and higher fabrication cost [33]. As a solution to this issue, additive manufacturing gained attention as it has undoubtable superiority, such as design freedom and short lead times [34]. Extensive research in this area implicates that achievable characteristics of Ti6Al4V are satisfying [35] and could even be superior for certain characteristics, like ductility [36]. 

The surface quality of CM components is usually higher than those fabricated via AM techniques and can be used in as-fabricated conditions. Ti6Al4V and other difficult-to-machine materials are usually shaped using optimized machining process parameters [37,38]. However, recent reports suggest the rapid development of AM techniques, which resulted in a sales increase of 27.2% for AM products when comparing 2023 to 2022. Continued advancements are expected in AM technologies, including speed, precision and quality of the surface [39,40]. This could also impact the titanium parts fabricated directly by additive manufacturing technology. Even though they already seem like good-quality products after manufacturing, these parts are not ready for service in their as-built state. Certain challenges and limitations are still associated with AM, including issues related to part quality or post-processing. Depending on the final application, each AM process requires one or more post-processing steps, according to the 2023 Wohlers report [40]. Additional surface-modifying treatment processes are needed due to surface defects and discontinuities in their surface layer. However, the specifics of the complex design of AM structures make it usually impossible to finish their surfaces by machining operations. Therefore, shot peening is one of the most effective surface treatments for AM metallic components [41]. 

#### 2.2.2. Additive Manufacturing Methods for Ti6Al4V Components

**Figure 6:**
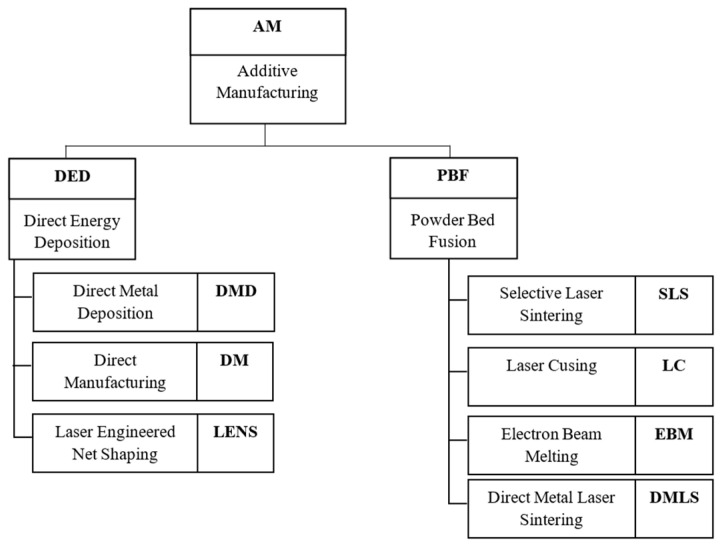
Additive manufacturing powder-based methods in accordance with ASTM F2792 based on [35].

Although the additive manufacturing methods are still developing, there are seven leading powder bed technologies as per the ASTM F2792 norm, as shown in Figure 6 [35].

The power of the laser and the feed rate of the powder have a direct impact on the homogeneity of an additively manufactured structure [42]. A Gaussian moving heat source could represent the laser beam power as in Equation (1):

(1) $$Q_{laser}=\frac{2Ap_{laser}}{\Pi r_{0}^{2}}\exp\left(\frac{-2{\left(x_{i}-x_{0}-v_{laser}t\right)}^{2}}{r_{0}^{2}}\right)$$

where *A* is the laser absorption coefficient, *P_laser_* is the laser power, *r*_0_ is the focus radius, *x*_0_ is the laser’s beginning beam location, *x_i_* is the position of laser focus and *v_laser_* is the laser’s scanning speed, and the heat transfer equation in accordance with Fourier’s law can be represented as (2) [43]:

(2) $$Q_{laser}=\rho_{m}C_{m}\left(\frac{\partial T}{\partial t}+u\cdot \nabla T\right)-\nabla \cdot \left(\lambda \nabla T\right)$$

where *ρ_m_* is the density, *C_m_* is the specific heat, *λ* is the base thermal conductivity and *u* is the fluid flow velocity.

The literature indicates that powder bed fusion (PBF) technologies are used for the industrial manufacturing of Ti6Al4V alloys [44,45]. In Table 5, a comparison of the properties between particular PBF AM technologies and direct energy deposition (DED) technology is presented.

#### 2.2.3. SLM—Selective Laser Melting

**Figure 7:**
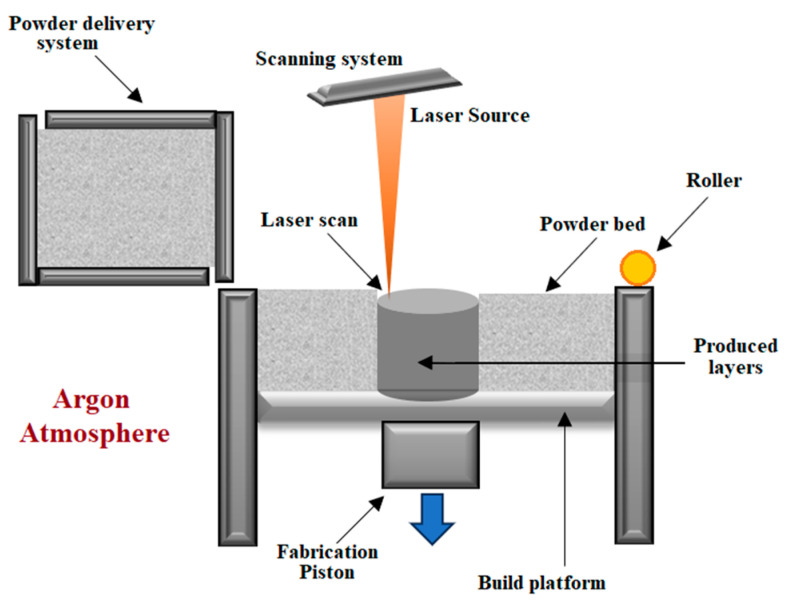
Diagram of the process of selective laser melting according to [20,61].

During the SLM process, a component is created by a laser beam, which interacts with Ti–6Al–4V powder, selectively melting layer by layer. After the powder delivery system spreads powder on a building platform, the particle of the material is heated, and after the application of appropriate power, it melts and forms a liquid pool. The molten pool consolidates and cools down quickly [59]. During the cooling process, the decomposition of β phase proceeds into diffusionless martensitic α′ [60]. Afterwards, the cross-section is scanned with each layer processed, the construction platform decreases in the fabrication space by the thickness of the new layer being applied and the process continues until the final product is formed [59]. Figure 7 shows a scheme of the SLM process.

The high reactivity of Ti-6Al-4V alloy demands that the process has to be carried out under an inert argon atmosphere [59]. SLM possesses several advantages, such as a high level of flexibility, a high efficiency of material use and the opportunity for the production of geometrically complex shapes of components close to the final product [62]. The main disadvantages are higher cost, large residual stresses caused by steep thermal gradients and defects such as those in conventional manufacturing like deformation [62], delamination [63], porosities [64] or even cracking of the parts in the form of hot cracking [65] or initiated by micro-sized defects [66].

#### 2.2.4. DMLS—Directive Metal Laser Sintering

**Figure 8:**
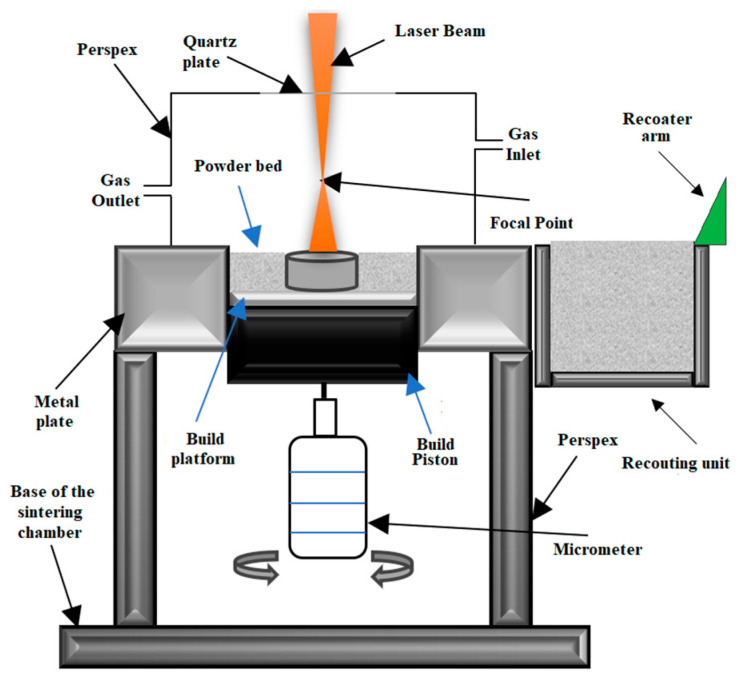
Schematic diagram of the DMLS process according to [71,72].

During the DMLS process, a laser power of low intensity is used to sinter the powder of Ti-6Al-4V alloy but not fully melt it like in the SLM process. The DMLS process is initiated by spreading a titanium alloy powder on the substrate, and then the laser beam scans over the entire surface to selectively fuse the powder. It is also possible that the laser beam partially melts powder in this process. Eventually, the powder bed solidifies to form a dense part [67,68]. The whole process is repeated after the platform is lowered by the distance of layer thickness until the final product is obtained [69]. The heat treatment and high cooling rate result in the creation of a dendritic or fully acicular martensite structure in Ti-6Al-4V titanium alloy [70]. Figure 8 shows the scheme of the DMLS process.

Observation of the DMLS technology is necessary as the occurrence of melting, re-solidification, shrinkage or heat transfer mechanisms such as convection or radiation is possible and makes the whole process more challenging [73]. Process defects such as high residual stresses, cracks and pores can also be observed [74].

#### 2.2.5. EBM—Electron Beam Melting

**Figure 9:**
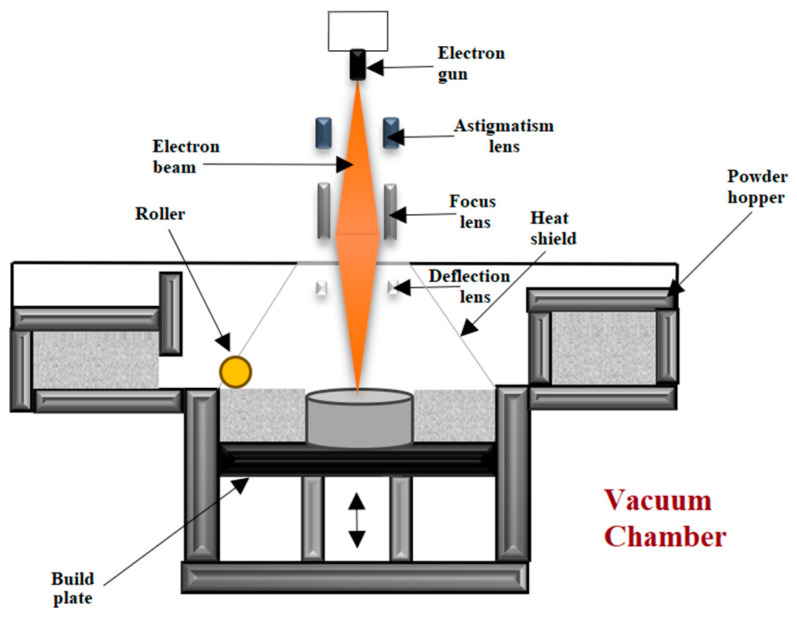
Schematic diagram of the EBM technique according to [79,80].

During the EBM process, an electron beam or focused laser is the heat source applied to melt the titanium powder. Layers are manufactured as follows: spreading the titanium powders on a base plate, preheating the powder, sintering the powder with a defocused beam, melting the powders by using a focused beam and then decreasing the building platform by the thickness of the layer [75,76]. Using the electron beam requires the powder to be sintered, or, in a different manner, the electrostatic forces lead to clouds of charged particles in the build chamber [77]. The whole EBM process is performed under a vacuum environment. This implies that highly reactive materials, such as titanium alloys, can be fabricated using the EBM technology without oxidation and contamination of the material throughout the process [78]. Figure 9 shows the scheme of the EBM process.

The defects after the EBM manufacturing process are similar to the defects after the use of other AM technologies. Different types of defects after EBM production of Ti-6Al-4V titanium alloy have different sources in the process [81]. The respective defect types exhibit differences in size and shape, which can impact the crack initiation process. A lack of fusion is also a common anomaly and is an effect of the under-melting of powder by the laser beam [82,83,84]. On the other hand, porosity is an outcome of the over-melting of the material and the vaporization of the titanium metal to some degree [84,85]. A further defect type is gas pores which can originate from the fabrication process or powder. Many defects can be avoided or minimized by appropriately selecting processing parameters; however, gas pores are considered to be inevitable [86]. The population of these anomalies in the EBM process has a direct influence on the fatigue life of Ti-6Al-4V titanium alloy [87].

#### 2.2.6. DED—Direct Energy Deposition Processes

**Figure 10:**
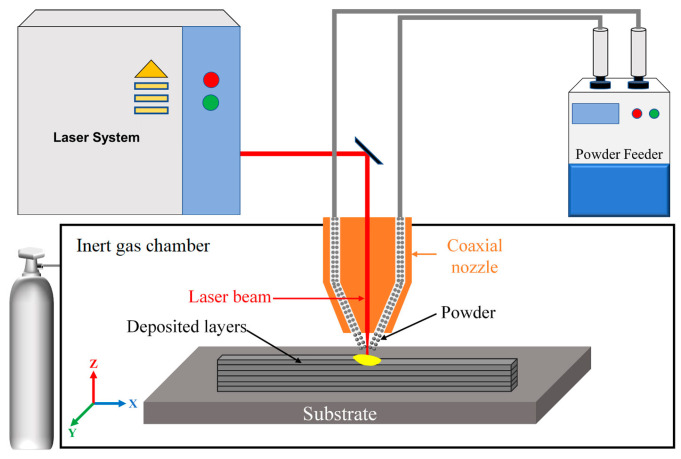
Schematic diagram of the DED process using laser beam [95]. Copyright Elsevier, 2024.

During direct energy deposition (DED), a stream of metallic powder or wire is fed into a melt pool that is created by a laser beam and melts as it is deposited [88]. This varies from powder bed fusion technologies (PBF) where thermal energy is used to selectively fuse regions of a powder bed. DED processes are typically used on existing parts of arbitrary geometry with a relatively high deposition rate [89]. The electron or laser beam creates a molten pool on the surface during the DED process. Then, the material transfer is conducted using a nozzle (laser as powder and beam as wire). The nozzle and the beam move along the path determined by the CAD data. The consecutive layers are melted and frozen on each other until the process is completed. The whole process takes place in a chamber filled with inert gas when the laser method is used or in a vacuum environment when an electron beam is used [75,90,91,92,93,94]. The scheme of building layers in DED technology using a laser is shown in Figure 10.

It has been well established that products of DED technology cannot be completely free of defects (i.e., pores, un-melted powder and lack of fusion (LOF)). LOFs are developed when the molten material in one layer does not completely fill the space between each DED pass, forming crack-like features perpendicular to BD [96,97,98,99]. Other typical defects after the DED process are shrinkage, residual stresses and deformations after local temperature differences occur, which means post-fabrication machining is often required. Utilizing wires and metallic sheets as printing feedstock material in DED processes usually leads to more defects, lower geometry precision, high surface roughness and limitations for the production of complex shapes when compared to the use of powder feedstock in PBF technologies. However, better static and dynamic mechanical properties are often obtained in the DED-deposited condition when compared to PBF-deposited parts [100,101,102].

## 3. Post-Process Treatments Applied to Modify Ti6Al4V Surface Layer Properties

**Figure 11:**
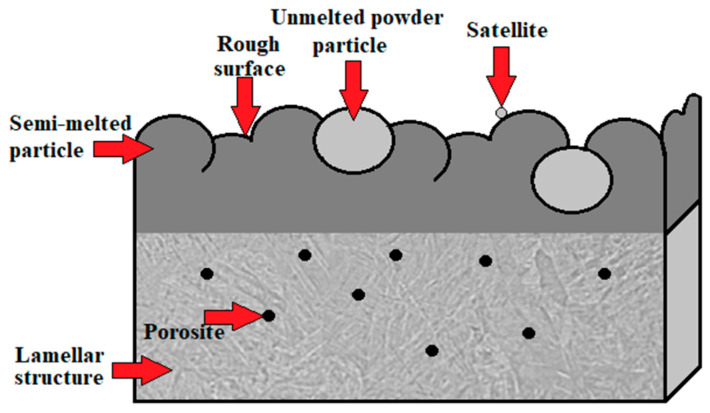
Schematic illustration of surface defects and anomalies after AM [105,106,107].

**Figure 12:**
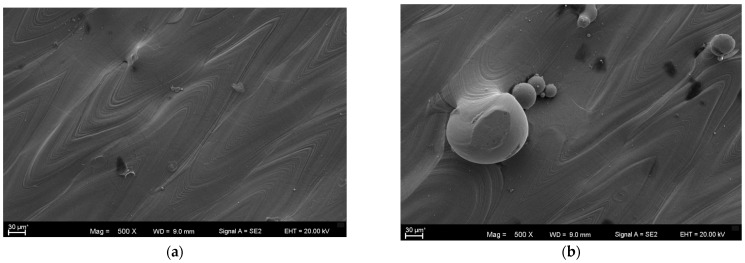
Typical surface defects and discontinuities of an AM surface of Ti6Al4V alloy: (**a**) collapse of the welding pool; (**b**) partially melted titanium powder [108].

Taking into account all defects arising after the fabrication process discussed in Section 2.2.3, Section 2.2.4, Section 2.2.5 and Section 2.2.6, after the use of each of the commonly used AM technologies, the conclusions that come to mind are that titanium alloy components fabricated directly by additive manufacturing are usually not ready for service in their as-built state due to technology shortcomings. To overcome these shortcomings, the AM parts have to be subjected to post-processing treatment, including support material removal, surface finishing, coloring, coating and heat treatment. Heat-treatment standards for conventionally manufactured metal parts and alloys are not created or adapted for additively manufactured products, and the obtained properties may differ from the desired properties in many cases depending on the initial microstructures. That is why it is crucial to determine the optimal parameters for the post-processing treatment as this not only improves the properties of these materials but also is beneficial in reducing the cost of the process [103]. However, the application of optimal 3D-printing parameters recommended by the producers does not avoid the exhibition of surface defects, anomalies and undesired residual stresses in Ti-6Al-4V products. These lead to unsatisfactory properties for their application and are a justification for the use of surface finishing treatments like the shot peening process [104]. The scheme of a typical surface layer after AM is shown in Figure 11, and SEM microphotographs, illustrating the morphology of a specimen’s surface after the use of the AM technology of DMLS, are shown in Figure 12.

### 3.1. Shot Peening

**Figure 13:**
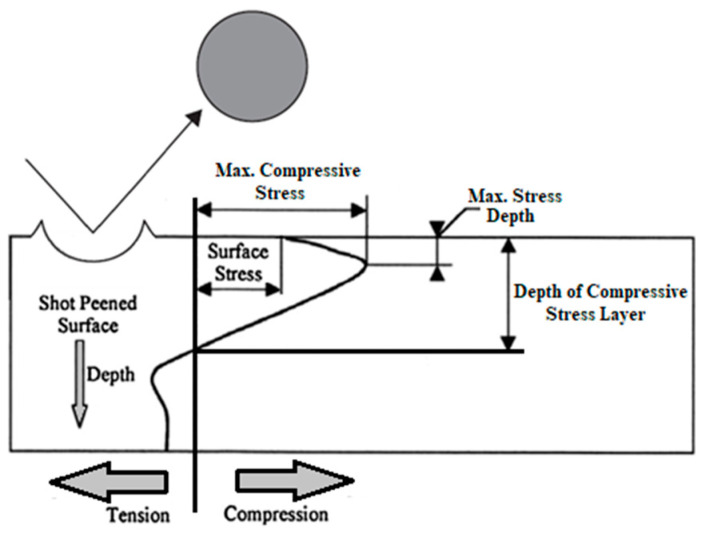
Effects of shot peening process according to [5,117]. Arrows indicate the direction of tension and compression stresses.

**Figure 14:**
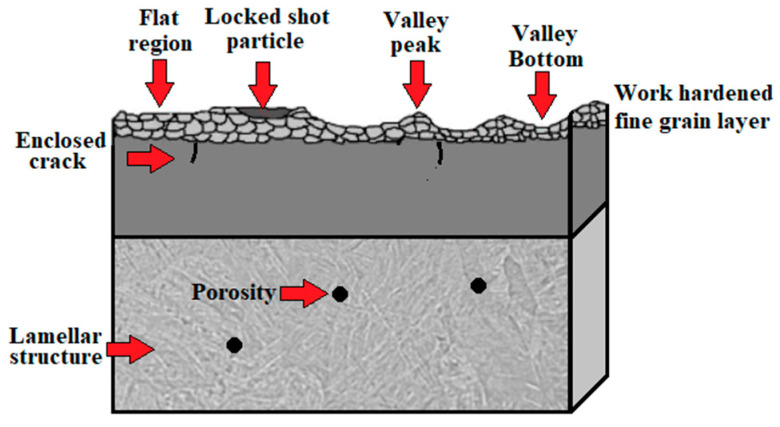
Schematic illustration of surface layer after shot peening treatment [105,106,107]. Arrows mark the most important features.

One of the most popular methods of modifying the surface layer is shot peening (SP). Shot peening is a mechanical surface treatment performed by the repeated impact of shots at high impact velocities onto the surface of a material that causes the plastic deformation of the material [109]. The achieved rate of surface layer plastic deformation protects metallic structures from fatigue [110,111,112], corrosion [113] and tribological wear [41]. It also has an impact on important mechanical properties of the material such as surface roughness [114], hardness [115] and residual stresses [116]. The scheme of the effect of the shot peening process is shown in Figure 13, and the modified surface layer structure obtained after the process is shown in Figure 14.

The overall favorable outcomes of the shot peening process can be summed up according to [99] as follows:

- Grain refinement;
- Increase in dislocation density;
- Formation of passive layer;
- Decrease in porosity;
- Formation of compressive residual stresses.

**Figure 15:**
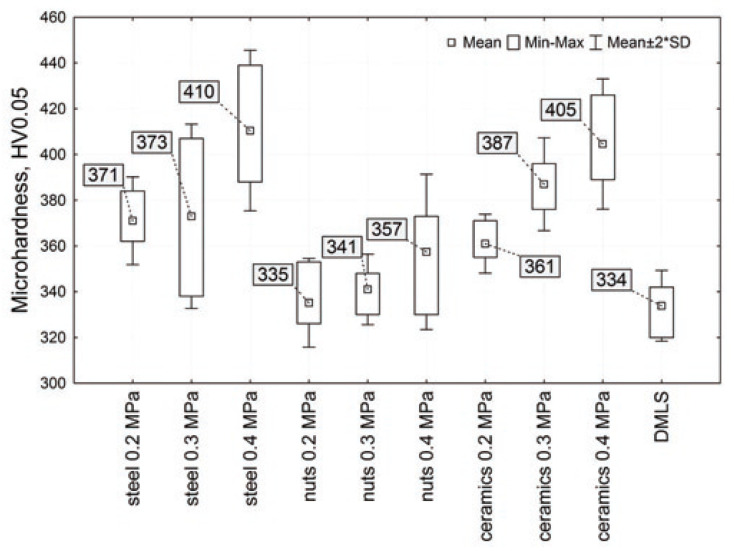
The hardness variation at different applied loads and shots for shot peening treatment of Ti-6Al-4V alloy [118].

According to the collection and the research of AM DMLS-manufactured samples of Ti-6Al-4V carried out by R. Żebrowski, M. Walczak and their team [21,106,108,118,119] for biomedical applications of titanium implants with complex shapes, the main advantage of shot peening is the improvement of working parameters. There is a relevant increase in hardness after that type of process from approximately 10% up to 25% for the highest peening pressure of 0.4 MPa, as shown in Figure 15 [118]. In comparison to subtractively manufactured Ti-6Al-4V, a similar increase was obtained for AM according to [120], from approximately 10% to 25% for the higher peening pressure of 0.5 MPa, as shown in Table 6.

The explanation for the surface hardness increase could be the creation of a nanocrystalline layer after shot peening treatment on the surface of the peened specimen which leads to the effect of material strengthening [121,122].

**Figure 16:**
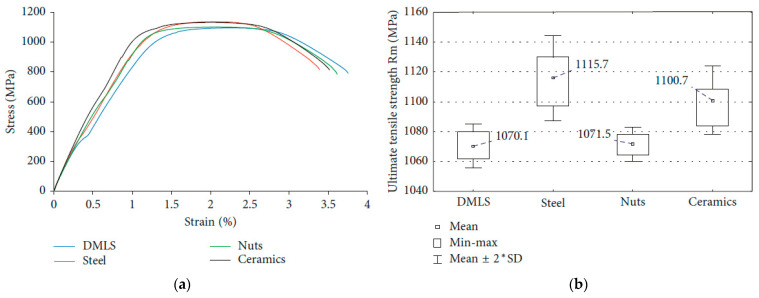
Tensile test: (**a**) stress–strain curves of various shot-peening-treated specimens; (**b**) ultimate tensile strength [106].

Compared to the hardness test, a similar effect of an increase was achieved for a group of specimens in an ultimate tensile strength test, which means that Ti-6Al-4V demonstrates similar correlations between hardness and UTS [106]. The results of tensile tests are shown in Figure 16.

**Figure 17:**
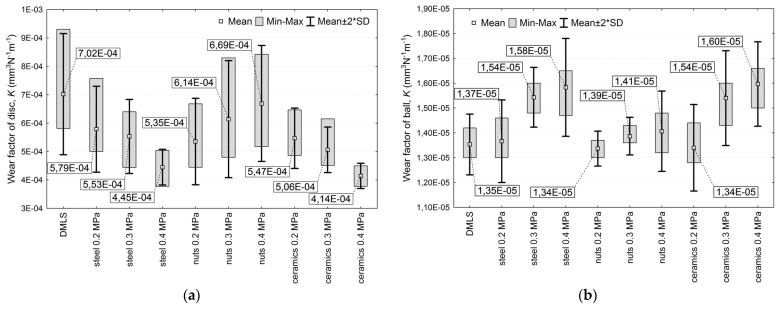
Illustration of wear factor K for tested Ti-6Al-4V additively manufactured (DMLS) before and after shot peening using different shots and peening pressures: (**a**) surfaces; (**b**) Al_2_O_3_ counter-bodies [108].

In terms of the wear and tribological performance of Ti-6Al-4V, there is a decrease in the friction coefficient for surfaces. There are changes associated with the use of balls that are made of Al_2_O_3_ as the counter-body. A comparable friction coefficient was obtained for soft surface subjection. The friction coefficient for some surfaces increased, as shown in Figure 17 [108].

**Figure 18:**
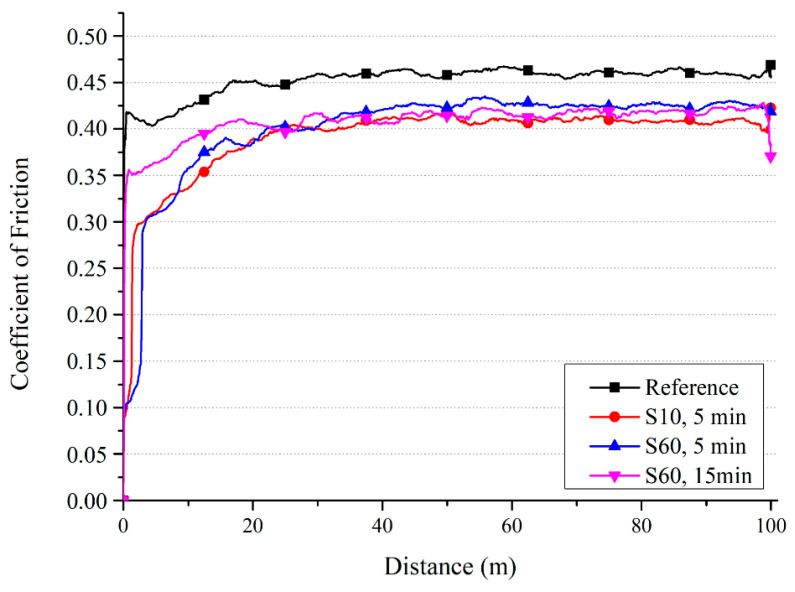
Characteristic of coefficient of friction to a sliding distance for conventional Ti6Al4V depending on shot peening treatment [123]. Copyright MDPI, 2020.

A similar decrease in COF was obtained for conventionally manufactured Ti-6Al-4V according to [123], as shown in Figure 18.

The signs of wear of Ti-Al-4V are typical for metallic materials with significant hardness and high ductility. The prevailing wear mechanism, which relies on abrasion and groove forming, is caused by the presence of the β phase. The plasticity of the β phase is higher than the plasticity of the α phase, which contributes to locally increased plastic deformation, as confirmed by Faria et al. [124]. The wear mechanisms for AM and SM of Ti-6Al-4V after shot peening are similar as they both depend on the uniformity and quality of the surface layer, which was confirmed by Airao et al. [125]. After shot peening, comparable structures are obtained for both AM and SM.

**Figure 19:**
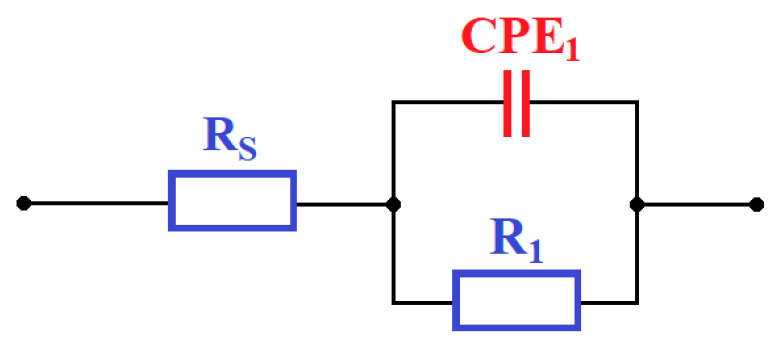
Equivalent circuit used to fit electrochemical impedance spectroscopy (EIS) spectra, based on work [119].

**Figure 20:**
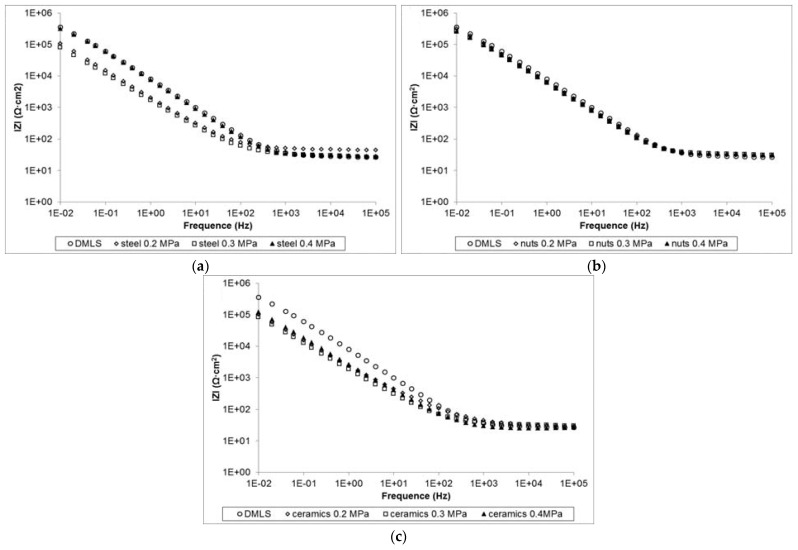
Bode plot characteristic of impedance module vs. frequency for surface modified (**a**) by means of steel shot, (**b**) by means of nutshells and (**c**) by means of ceramic beads [119].

The equivalent electric circuit for experimental data is shown in Figure 19. This circuit is constituted by a constant phase element (CPE_1_), used to simulate a non-ideal behavior of the condenser due to the passive oxide layer, the electrolyte resistance (Rs) and the charge transfer resistance (R1). In terms of corrosion behavior, there is a decrease in surface impedance (Figure 20). The simulated values are included in Table 7. From an analysis of the results, it appears that the value of R1 resistance decreases with the increasing pressure of the shot peening process for all modified surfaces. A high value of R1 resistance results in higher corrosion resistance. 

The impedance spectra in Figure 20 represent the impedance module versus frequency and display impedance values after shot peening close to those for untreated specimens. The obtained results after shot peening are high and situated in the range of 10^5^ ÷ 10^6^ Ω·cm^2^ at low frequencies, which means that these surfaces are sufficient for bioengineering purposes with enough corrosion resistance in body fluids [121,126,127]. The electrochemical properties of Ti-6Al-4V after the shot peening process are shown in Table 8.

Like the increase in surface hardness, the increase in corrosion resistance can be explained by the spontaneous creation of a nanocrystalline layer after shot peening treatment. This passive layer created on Ti-6Al-4V surgical alloys is stable in Ringer fluid solution and is rich mainly in amorphous TiO_2_ [121,122]. However, more factors should be taken into account. The disorientation of the topography is reported to play a crucial role in corrosion resistance [128], as can be seen in Table 6. Mechanically polished surfaces exhibited favorable electrochemical parameters. They are favorable due to low roughness values (especially for the Sa parameter) and the lack of structural discontinuities in the surface layer created in the course of the DMLS process [118]. Comparing AM to CM [129] indicates that the corrosion behavior for polished surfaces after shot peening treatment could be more promising for 3D printed surfaces than for conventional ones.

Surface roughness also influences coating adhesion factors such as droplet impact, wetting and solidification [130]. Other factors that have a direct impact on coating adhesion are stiffness and hardness. Comparing DMLS technology and conventional manufacturing indicates that the formation of a martensitic structure during the use of additive manufacturing methods like the use of DMLS technology makes the substrate stiffer and harder, with approximately 20% higher nanohardness and elastic modulus of the surface layer than an alloy fabricated by conventional methods. This outcome is affected by the phase composition of the Ti-6Al-4V substrate as well as the phase composition of coatings. The DMLS alloy had a martensitic α′ phase with a hexagonal lattice (a = 0.2937 nm, c = 0.4652 nm), and its conventional counterpart had a two-phase structure of α + β with a hexagonal lattice of the α phase and cubic lattice (a = 0.3309 nm) of the β phase. The AlTiN coating consisted of the α phase and Al_0.5_Ti_0.5_N phase with a cubic crystal lattice (a = 0.419 nm). The TiAlN coating consisted of the α phase and Al0.35Ti0.65N phase with a cubic crystal lattice (a = 0.41805 nm). The fitness of E_coating_/E_substrate_ for PVD nitride coatings is shown in Table 9.

The results shown in Table 9 were obtained using the magnetron sputtering method, which was used for depositing PVD coatings. However, recently, the most employed method for forming coatings, films or layers on surfaces of titanium or its alloys has been plasma nitriding. According to Balasubramanian, ion plasma treatment for Ti-6Al-4V alloy forms a titanium nitride layer that displays not only better surface roughness characteristics and mechanical properties but also superior tribological properties when compared to non-treated specimens [131]. A comparison between ion-nitrided specimens and specimens not subjected to the plasma ion-nitriding process is shown in Table 10.

### 3.2. Other Peening Methods

There are various modern peening methods that could be applied instead of conventional shot peening. The modern peening methods are generally classified as follows [132]:

- Ultrasonic impact peening;
- Laser shock peening;
- Water jet peening;
- Oil jet peening.

Processes similar to conventional shot peening are surface nanocrystallization (SNC) treatments, which include ultrasonic-assisted surface mechanical attrition treatment (SMAT). SMAT is a derivation from the conventional shot peening process where the balls impact the surface in more random directions unlike in shot peening. There are three techniques used in SMAT. The first technique is based on the vibration of spherical shots using high-power ultrasound. The second type is based on mechanical vibration. The third type is based on a pneumatic assistance system [133,134]. In the literature, few investigations were carried out for the SMAT method for Ti-6Al-4V alloy for its dental implant and other biomedical applications. The found results are shown in Table 11 and Table 12.

### 3.3. Electropolishing

**Figure 21:**
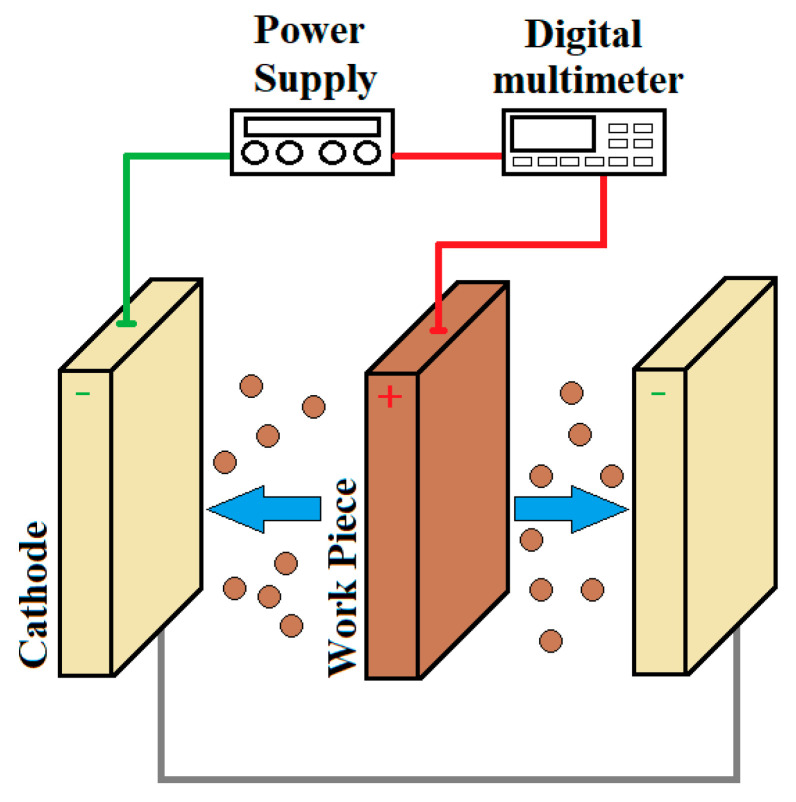
Schematic diagram of a typical system of an electrolytic cell for electropolishing according to [142,143].

**Figure 22:**
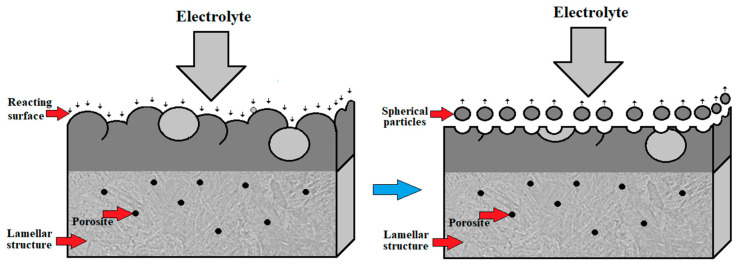
Mechanism of electropolishing of Ti6Al4V object after AM according to [142,144]. Red arrows mark the most important features. Black arrows indicate the changes which occur on the surface layer due to electrolyte action.

Electropolishing (EP) is an extremely efficient electrochemical surface finishing technique that does not cause any deterioration in metal component structures. During electropolishing, the material works as an anode and is connected to the positive electrode. The anode metal is oxidized into metal ions due to the loss of electrons. Then, it dissolves into the electrolyte, leading to the removal of surface materials in order to polish, passivate and deburr the metal parts [136,137,138,139,140]. This method does not cause mechanical interaction or damage or leave any residual stress [141]. A schematic example of a typical electropolishing setup and the mechanism of electropolishing for titanium alloys are shown in Figure 21 and Figure 22.

The electrolytes used in the electrochemical processes of electropolishing titanium and titanium alloys are as follows:

- Perchloric acid-based electrolytes, for instance, perchloric acid/acetic acid and perchloric acid/methanol/ethylene glycol systems [145,146];
- Perchloric acid-free electrolytes, such as methanol/sulfuric acid and ethylene glycol/NaCl [147,148];
- Deep eutectic solvents (DESs) such as ChCl [149,150].

In a comparison of mechanical polishing to electropolishing, surface layer characteristics [151] and corrosion behavior properties [152] are more favorable for the electropolishing of conventionally made Ti-6Al-4V. The literature [151,152,153,154] also indicates that surface roughness (Ra parameter) is affected by the time, current density and temperature of the process, and depending on parameter selection, roughness can increase (Table 13) [153] or decrease (Table 13 and Table 14) [151]. Temperature influences material roughness after electropolishing treatment, which is also shown in Table 13.

Although many previous investigations have reported the properties and electrochemical behaviors of traditional titanium alloys after electropolishing based on different bath components and process parameters, the special surface state of additively manufactured titanium alloys has limited their usage [155]. This can be ascribed to titanium alloys fabricated by AM often having incomplete melting powder, which generates an oxide film-like ceramic with high hardness and low chemical activity [156].

According to Zhang’s research [144] on electropolishing of additively manufactured Ti-6Al-4V specimens by selective laser melting, optimal electrochemical treatment is able to improve the roughness and impedance (Table 15) of titanium alloy.

## 4. Combination of Shot Peening and Electropolishing

**Figure 23:**
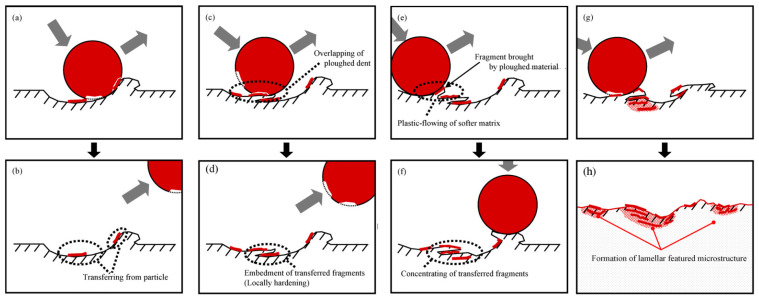
Embedment of shot particle in material surface layer, (**a**–**h**) shows the stadia of surface deformation due to peening, according to [122]. Copyright Elsevier, 2009.

For many fields of application, the properties contributed by the shot peening process might be beneficial, but they are overshadowed by the unfavorable characteristics of the roughened surface. An example of this is the automotive branch where reports suggest that shot-peened iron gears have poor performance in contact fatigue resistance due to the roughness of the surface layer [157]. Another issue that challenges its successful application in some fields is the potential presence of locked shot particles on the shot-peened surfaces introducing potential crack nucleation sites (Figure 23). In biomedical applications, these particles could be responsible for early implant failure [158]. 

**Figure 24:**
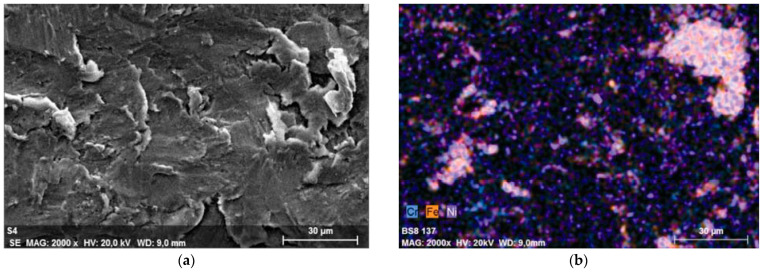
Formation of lamellar featured microstructure on Ti6Al4V surface after SP with CrNi shots: (**a**) shot inclusions on specimen surface; (**b**) mapping of elements originating from steel shot [119].

The topography of a surface-treated Ti-6Al-4V alloy is modified by shot grains penetrated into the surface layer. The effect of this process is visible in Figure 24. The high kinetic energy of the shot causes the penetration into the surface layer of the printed subject which results in structural discontinuity and the formation of a lamellar structure [119].

In this regard, it is necessary to control the peening processes to remove embedded remains of peening media in order to meet the requirements of a smooth and defect-free surface for certain applications. This suggested method could provide further improvement of material properties such as corrosion resistance and wear [159].

Research performed by M. Kiel and J. Szewczenko’s team [160,161,162,163] on combining shot peening and electropolishing for conventionally made Ti-6Al-4V showed promising results. The effect of combining shot peening and electropolishing on corrosion behavior is shown in Table 16.

In addition, this literature review indicated a research gap since, as far as the authors’ knowledge goes, there are no studies describing a hybrid treatment consisting of a combination of shot peening and electropolishing methods on additively manufactured objects, including Ti6Al4V titanium alloy. A few reports suggest that electropolishing reduces the presence of embedded fragments after shot peening treatment and smooths the surface of peened samples, which could be beneficial for the properties of titanium alloys. A research area in this direction could include, for instance, the application of EP treatment after SP or EP treatment followed by SP. In addition, optimizing hybrid process parameters such as different peening times, intensities or shot sizes in the SP process and different polishing times, types of electrolytes and voltages in the EP process would also be required. This literature gap leads to the scope of future work about the effects of shot peening and combined peening and electropolishing on the wear and corrosion resistance performance of additively manufactured titanium alloys.

## 5. Summary

This review paper clarifies the effects of shot peening and electropolishing treatment on the properties of both additively and conventionally manufactured Ti6Al4V alloys. The following conclusions are drawn:

1. This literature review confirms that Ti-6Al-4V fabricated using conventional methods such as casting, plastic forming or machining processes has an α + β dual-phase microstructure. Additive manufacturing methods change the microstructure. Due to the rapid heat exchange during heat treatment, the bimodal microstructure transforms into a lamellar microstructure consisting of two phases: α′ + β. This microstructure difference is crucial for the operational performance of fabricated parts because it seriously affects the mechanical properties of CM and AM components.
2. Conventionally manufactured Ti6Al4V alloy components are fabricated using a broad range of well-known fabrication technologies such as casting, forging and machining. In contrast, AM processes for Ti6Al4V alloy are mostly limited to two main fabrication techniques, i.e., direct energy deposition (DED) and powder bed fusion (PBF). Both CM and AM components can be further heat-treated and processed via surface engineering techniques.
3. Usually, surface treatment processes such as shot peening (SP) and electropolishing (EP) of titanium alloys are required to achieve the required surface quality. Both conventionally and additively manufactured components require adequate surface finishing to obtain the required surface layer roughness or mechanical properties. A literature survey indicates that shot peening is a standard procedure used for CM and AM titanium alloys. On the other hand, minimal attention has been paid to studying the electropolishing of additively manufactured Ti6Al4V samples.
4. Titanium parts fabricated by additive manufacturing technology, even though they seem like good-quality products after the manufacturing process, are usually not ready for service in their as-built state. Each AM process requires one or more post-processing steps, depending on the final application. The most method popular in the industry for improving the quality of the surface of AM components is shot peening.
5. Shot peening post-processing of AM components improves the surface quality and mechanical properties of the components. Depending on the applied parameters, the process can reduce surface roughness, minimize the surface nonuniformities, harden the surface layer and refine the grain size of Ti6Al4V alloy, all of which are beneficial for the further operational performance of components, i.e., improving resistance to corrosion, wear, etc. On the other hand, electropolishing improves surface quality by reducing surface roughness and improving corrosion resistance without deterioration in the structure of metallic components. There are many reports on the properties and electrochemical behaviors of traditional titanium alloys after electropolishing based on different bath components and process parameters, but the special surface layer state of additively manufactured titanium alloys makes new investigations on this issue necessary.
6. Finally, this review paper indicates the information about the effects of combining the peening process and electropolishing of additively manufactured titanium alloys, which seems to be a drawback in the state of the art. The synthesis of these two processes could provide the required surface layer quality free of surface nonuniformities characteristic of additively manufactured alloys. So, investigating the effect of this hybrid surface treatment, i.e., shot peening and electropolishing, of additively manufactured Ti6Al4V titanium alloy on surface layer properties is a promising direction for our group’s future research scope.

## Figures and Tables

**Table 1:** Comparison of physicomechanical properties of popular biomedical materials, namely Ti6Al4V titanium alloy, 316 L stainless steel and CoCrMo alloy, with natural human bone [8,9,10,11].

Property	Natural Human Bone	Ti6Al4V Alloy (Wrought)	316L Stainless Steel (Cast)	F75CoCrMo Alloy (Cast)
Density (g/cm^3^)	1.5–2	4.4	8.0	8.8
Tensile modulus of elasticity (GPa)	-	830–1070	205	500–1500
Yield strength (MPa)	130–190	920–1140	515	900–1800
Ultimate tensile strength (MPa)	10–30	100–110	195–205	200–230
Elongation (%)	-	10–15	10–40	4–13

**Table 2:** Chemical composition of Ti-6Al-4V powder used in AM [17].

Al	V	Fe	O	C	Ti	Others
5–6.75	3.5–4.5	≤0.25	0.14–0.16	≤0.02	Bal.	0.4

**Table 3:** The development of Ti6Al4V microstructure under different cooling media [23].

Phase Transformation Region	Temperature Range (°C)	Microstructure in Various Cooling Media		
		Water	Air	Furnace
α + β	700–950	A mixture of α and β structures, with more volume of α structures	Primary α, with grains having α + β lamellar structure	Primary α phase with intergranular β phase observed for all temperatures α phase on the phase boundary and a transition from β to α on the grain boundary. The grains were observed to have α/β lamellar structure
β	950–1100	Martensite microstructure consists of a fine acicular α phase with grain boundaries consisting of β phase	Partial martensitic microstructure, there exists an incomplete transition from β to α phase on grain boundaries	

**Table 5:** Comparison of mechanical properties of Ti6Al4V after manufacturing with particular AM technologies [30,46,47,48,49,50,51,52,53,54,55,56,57,58].

AM Technology	Specimen Orientation and Ref.		Mechanical Properties				
			E [MPa]	Microhardness	YS [MPa]	UTS [MPa]	A [%]
DED	XZ	[46]	-	-	522	797	1.7
	XY	[46]	-	-	892	911	6.4
	XZ	[30]	-	-	945	1041	14.5
	XZ	[30]	-	-	970	1087	13.6
	XY	[47]	-	-	960	1063	10.9
SLM	XZ	[48]	115	-	978	1143	11.8
	ZX	[48]	119	-	967	1117	8.9
	XY	[48]	113	-	1075	1199	7.6
	XY	[49]	-	394 HV	1052	1136	2.92
	XY	[50]	-	370 HV_0.3_	1273	1421	3.2
	XZ	[50]	-	390 HV_0.3_	1150	1246	1.4
	XY	[51]	-	350 HV	-	1137	9.10
EBM	XY	[52]	118	321 HV	830	915	13.1
	XY	[53]	114	35 HRC	830	914	13.1
	XY	[50]	-	315 HV_0.3_	846	976	15.0
	XZ	[50]	-	340 HV_0.3_	845	972	14.2
	ZX	[54]	-	-	869	965	-
DMLS	ZX	[54]	-	380 HV	1017	1096	16
	ZX	[55]	111.9	871 HV10	1086	1121	16.9
	XY	[56]	110	400–430 HV	1140	1290	7
	XY	[57]	-	-	990	1045	14
	ZX	[58]	108.0	-	982	1080	14.3
	XZ	[58]	108.7	-	980	1072	14.1

**Table 6:** Hardness of conventionally made Ti-6Al-4V with different surface treatment conditions [120].

	Name of Shot Peening Medium (Type of Medium)			
	Untreated	SUS100 (SUS304)	SUS400 (SUS304)	FHB 80 (SiO_2_)
Peening pressure	-	0.5 MPa		
Hardness	371 HV	420 HV	440 HV	470 HV

**Table 7:** Results of electrochemical impedance spectroscopy (EIS) studies [119].

Conditions		R_s_ (Ωcm^2^)	R_1_ (Ωcm^2^)	CPE_1_	
				Y_1_ (S/cm^2^)	N_1_
Untreated		27.79	898.02 × 10^1^	25.87 × 10^−6^	0.89
Steel CrNi	0.2 MPa	31.45	1278.40 × 10^2^	102.20 × 10^−6^	0.84
	0.3 MPa	29.59	1010.44 × 10^1^	128.01 × 10^−6^	0.82
	0.4 MPa	29.11	528.75 × 10^1^	26.26 × 10^−6^	0.91
Nuts	0.2 MPa	30.21	925.98 × 10^1^	30.71 × 10^−6^	0.88
	0.3 MPa	32.38	555.34 × 10^1^	33.00 × 10^−6^	0.88
	0.4 MPa	32.69	539.05 × 10^1^	33.44 × 10^−6^	0.88
Ceramic Beads	0.2 MPa	32.02	4970.23 × 10^3^	95.53 × 10^−6^	0.77
	0.3 MPa	32.31	1096.42 × 10^3^	119.57 × 10^−6^	0.81
	0.4 MPa	25.32	3120.03 × 10^1^	83.06 × 10^−6^	0.82

**Table 8:** Electrochemical corrosion tests in 0.9% NaCl for various peened surfaces of Ti-6Al-4V [118].

Conditions		Current Density, I_corr_ (mA/cm^2^)	Potential, E_corr_ (mV)	Polarization Resistance R_p_ (kΩcm^2^)
Untreated		0.064	−318.6	2291
Untreated mechanically polished		0.067	−141.1	328.5
Cr-Ni Steel Shots	0.2 MPa	0.421	−173.5	210.5
	0.3 MPa	0.561	−207.4	138.8
	0.4 MPa	0.682	−337.1	81.2
Nuts	0.2 MPa	0.124	−106.6	346.5
	0.3 MPa	0.275	−228.5	367.4
	0.4 MPa	1.469	−279.2	349.5
Ceramic Beads	0.2 MPa	0.026	−123.8	170.8
	0.3 MPa	0.045	−151.4	206.2
	0.4 MPa	0.063	−174.3	432.8

**Table 9:** Mean hardness and elastic modulus of surface layer and their ratio for DMLS and conventional samples (instrumented indentation test according to PN-EN ISO14577-1 standard) [21].

Substrate: Ti6Al4V	Coating					
	-		AlTiN		TiAlN	
	DMLS	Conv.	DMLS	Conv.	DMLS	Conv.
S_a_ [µm]	0.014	0.040	0.027	0.038	0.028	0.053
H_IT_	5.7 ± 0.2	4.8 ± 0.4	25.0 ± 4.6	26.1 ± 4.3	23.6 ± 3.4	23.2 ± 3.3
E_IT_	137.0 ± 4.1	114.5 ± 4.7	518.7 ± 129.1	559.2 ± 117.3	411.4 ± 45.8	503.5 ± 99.4
H_coating_/E_coating_	-	-	0.048	0.047	0.057	0.046
H^3^_coating_/E^2^_coating_	-	-	0.059	0.057	0.078	0.049
E_coating_/E_substrate_	-	-	3.79	4.88	3.00	4.40

**Table 10:** Surface coating characteristics after ion plasma treatment [131].

Substrate: Ti6Al4V	Coating: TiN (Top); Ti_2_N (Bottom)		
	Average Hardness Value [VHN]	R_a_ [µm]	Wear [µm]
Non-nitrided specimen	311	0.225	493
Plasma nitrided specimen	364	0.195	355

**Table 11:** Properties of AM Ti6Al4V after surface crystallization effect in SMAT [135].

Sample Treatment	Hardness [HV]	YS (MPa)	UTS (MPa)
Untreated	345	888	1074
Heat treatment	370	903	1032
SMAT	380	973	1134
Heat treatment and SMAT	420	1016	1152

**Table 12:** Corrosion resistance of CM Ti6Al4V after surface crystallization effect in SMAT [121].

Sample Treatment	I_corr_ (mA/cm^2^)	E_corr_ (mV)	R_s_ (Ω)
Untreated	−330	0.052	13.83
SMAT (T = 15 min. Bead Diameter = 2 mm)	−275	0.035	38.82
SMAT (T = 20 min. Bead Diameter = 3 mm)	−235	0.022	19.96

**Table 13:** Surface roughness parameters (Ra) of Ti-6Al-4V titanium alloy after different electropolishing conditions based on [153,154].

Conditions	Ref	Current Density of Treatment (mA·cm^−2^)	Time [Min]	Temperature [°C]	Ra (nm)
Untreated	[153]	-	-	-	321
Electrochemical etching (Ethaline)	[153]	5	20	25	651
		10			967
		15			504
Grinding, electrochemical polishing (sulfuric acid solution)	[154]	190	8	7	7.4
				18	6.1
				25	6.4

**Table 14:** Comparison of roughness for mechanical and electropolishing treatment [151].

Roughness	Microstructure in Various Cooling Media					
	Untreated	Electropolishing 100 s	Electropolishing 200 s	Electropolishing 300 s	Mechanical #1000SiC	Mechanical #1500SiC
Micro [nm]	120.05 ± 7.89	58.72 ± 3.68	12.63 ± 0.81	10.33 ± 1.14	98.30 ± 3.79	86.42 ± 2.05
Macro [µm]	2.34 ± 0.07	1.68 ± 0.02	0.75 ± 0.05	0.68 ± 0.03	2.04 ± 0.03	1.82 ± 0.09

**Table 15:** Surface roughness, weight loss and impedance parameters after different EP times for Ti-6Al-4V alloy according to [144].

Sample No.	0	1	2	3	4
Polishing time (min.)	untreated	5	10	15	20
Weight loss [%]	-	5.98	10.82	14.76	16.29
R_a_ [µm]	6.33	2.01	1.63	1.132	1.72
R_L_ (Ω·cm^2^)	-	20.69	16.44	21.49	23.29
Q_c_ (F·cm^−2^)	-	2.016 × 10^−2^	2.507 × 10^−5^	5.625 × 10^−5^	4.656 × 10^−5^
R_c_ (Ω·cm^2^)	-	758.40	8.56	12.72	23.26
Q_d_ (F·cm^−2^)	-	-	2.663 × 10^−6^	3.724 × 10^−6^	2.051 × 10^−6^
R_t_ (Ω·cm^2^)	-	-	−7.152 × 10^5^	1.014 × 10^6^	5.128 × 10^5^
Chi-squared (X^2^)	-	2.02 × 10^−3^	1.48 × 10^−3^	7.97 × 10^−3^	1.59 × 10^−3^

**Table 16:** Corrosion behavior after different surface treatments of Ti-6Al-4V ELI alloy based on [160,161,163].

Surface Treatment Processes	Ref.	E_corr_ [mV]	I_corr_ [µA·cm^−2^]	R_p_ [kΩ·cm^2^]
Grinding	[160]	−95	0.050	557
Grinding, shot peening, mechanical polishing, sandblasting	[161]	−168	0.090	278
Grinding, shot peening, mechanical polishing, sandblasting, electropolishing	[161]	−450	0.040	720
Shot peening, sandblasting	[163]	−266	0.047	551
Shot peening, electropolishing	[163]	−95	0.053	489
Shot peening, mechanical polishing, electropolishing	[163]	−172	0.069	377

## Data Availability

No new data were created or analyzed in this study. Data sharing is not applicable to this article.
